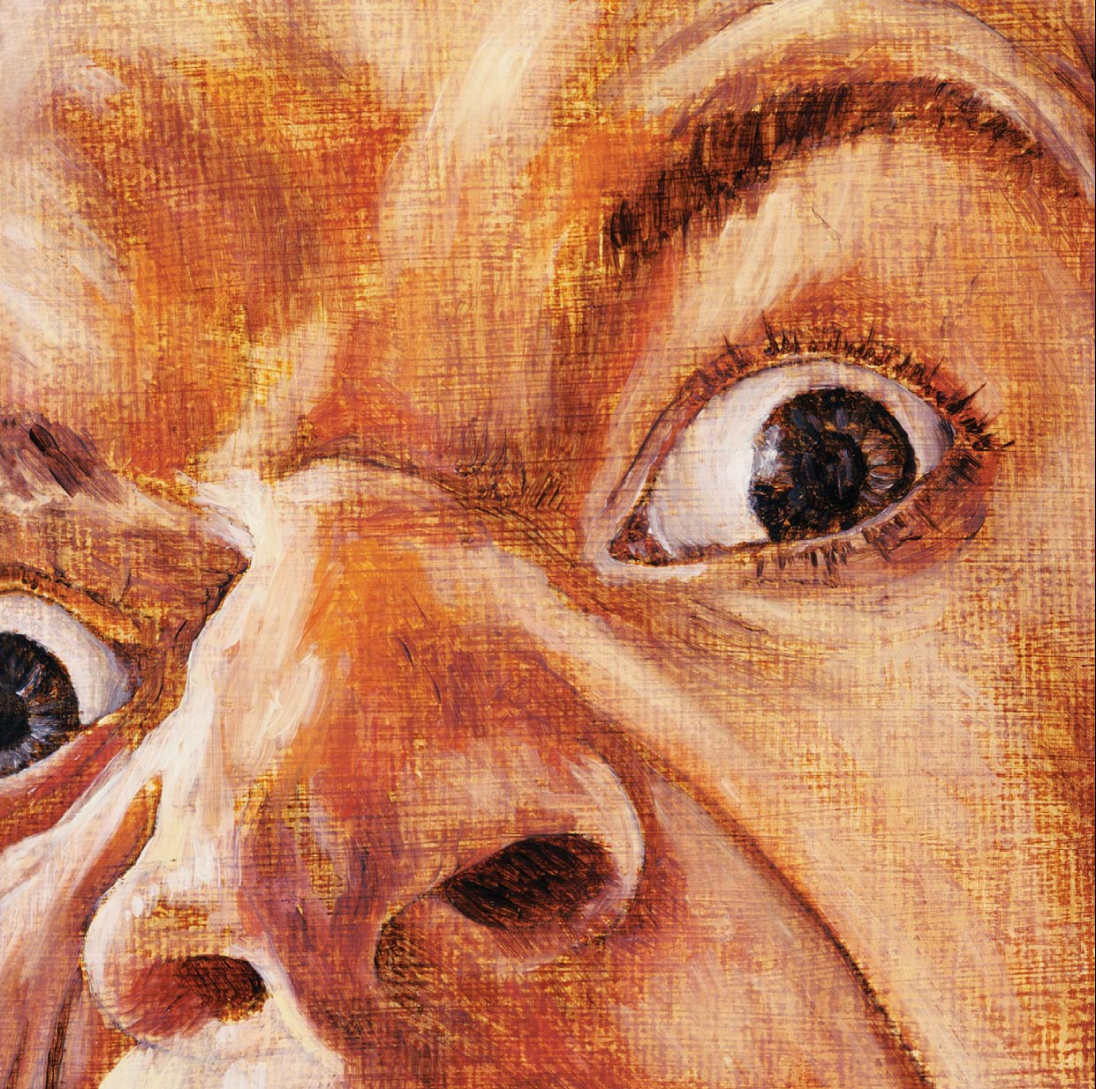# The Yuck Factor When Disgust Meets Discovery

**DOI:** 10.1289/ehp.116-a524

**Published:** 2008-12

**Authors:** Charles W. Schmidt

Imagine you lived in a drought-stricken area and were told that from now on your tap water would come from “recycled sewage.” Might the word “yuck” describe your gut response? If your answer is yes, then you’ve got lots of company. Most people instinctively reject fearsome or repugnant things, especially when those things are unfamiliar. If shared by masses of people, that collective repugnance can fuel a social force with the power to shape environmental and public policy.

The so-called yuck factor, a term coined by University of Pennsylvania bioethicist Arthur Caplan to describe the influence of instinctive responses against new technology, has a wide scope. In California, it’s derailed projects for converting wastewater into drinking water in several municipalities. It’s been cited in public opposition to foods from cloned animals and genetically modified (GM) crops. It’s even been named as a barrier to programs for trading carbon dioxide emission credits on the open market, says Åsa Löfgren, an economist at Göteberg University, Sweden, who points to widespread aversion to the notion that companies could buy rights to pollute.

Generally speaking, “yuck factor” has become a catchall phrase to describe technophobic sentiments that vary by what triggers them. The disgust elicited by drinking reclaimed wastewater, for instance, differs from the moral outrage induced by human cloning.

Meanwhile, science routinely generates technologies that—though they might initially be seen as repugnant—are also borne of real need. For instance, wastewater reclamation, the process by which sewage water is treated to augment drinking, industrial, and agricultural water supplies, responds to the growing problem of drought. In this case, the yuck factor—exacerbated perhaps by the use of terms such as “recycled sewage” and “toilet-to-tap”—stands in the way of a solution to dwindling water supplies that experts generally view as cost-effective and safe.

Galvanized by the yuck factor, opponents in Redwood City, California, delayed a wastewater reclamation project for nearly two years. And about six hours north, in Fountain Valley, a group dubbed the Revolting Grandmas led opposition to the Orange County Groundwater Replenishment System, which is the largest wastewater reclamation plant in the world. Responding to opponents’ demands, engineers now pump highly treated wastewater leaving the plant into an underground basin, where it filters through layers of sand and gravel before being piped to the homes and businesses that use it. Ironically, the water coming out of the basin isn’t as clean as the treated water going into it, according to an article in the 8 August 2008 *New York Times Magazine*—during its trip through the natural filters it picks up trace elements and contaminants that must later be removed by the water utility. The underground filtration step is taken, says director of recharge operations Adam Hutchinson, strictly to allay psychological concerns.

But the yuck factor could also be said to serve a useful purpose. Excrement does pose health risks, and the public is therefore wise to ask questions about the safety of drinking reclaimed wastewater. Likewise, genetic technologies have the capacity to fundamentally alter life as we know it, in some cases with uncertain benefits. By giving pause to technological progress, the yuck factor opens new opportunities for dialogue between scientists and the public. In some cases, that dialogue might show that a technology’s benefits outweigh the repugnance that goes with it. In others, it pushes scientists to make a better case for why a given technology should be pursued at all.

Given the influence wielded by instinctive responses to new technology, Caplan asserts that policy makers need to better understand these responses and take them seriously. “Savvy marketers and good advertising people know how to appeal to emotion, gut rationality, and visceral fears,” he says. “That’s what they’re selling—the manipulation of ‘yuck’—and more often than not, this is what determines who wins or loses in science policy debates. If you really want to overcome that, you have to become sophisticated about how experts manipulate emotion. And if you’re going to assess what’s admissible in terms of public policy, your argument has got to be better than to simply say ‘I don’t like it.’”

## Layers of Perception

In one sense, the yuck factor reflects disgust, says Paul Rozin, a professor of psychology at the University of Pennsylvania. Both humans and animals express disgust in similar ways, he adds, which suggests the reaction was conserved during evolution. “It produces a characteristic facial expression,” Rozin explains. “There’s a grimace, the lower jaw drops, the tongue sticks out, and the nose wrinkles. We’re not sure about its origins; it probably has to do with avoiding contagious illness.”

Intimately connected with disgust are accompanying feelings of fear, Rozin says. Together, those reactions can be hard to overcome, adds Brent Haddad, a professor of environmental studies at the University of California, Santa Cruz. In an unpublished survey of 2,500 people questioned in public areas, Haddad and Rozin found that 13% of respondents claimed they would never drink highly treated waste-water despite a broad scientific consensus that it can be done safely.

Savvy marketers and good advertising people know how to appeal to emotion, gut rationality, and visceral fears. That’s what they’re selling—the manipulation of “yuck”— and more often than not, this is what determines who wins or loses in science policy debates.

–Arthur Caplan, University of Pennsylvania

On a more cognitive level, repugnance can also be triggered by perceived violations of morality. Writing in the April 1999 *Journal of Personality and Social Psychology*, Rozin and colleagues proposed that violating moral codes relating to divinity and sanctity can provoke reactions of disgust. Studies that involve creating and destroying human embryos have elicited such reactions. Similarly, using biotechnology to alter the structure and characteristics of crop genes—for instance by mixing animal and plant DNA—might be viewed as tampering with divine creation.

What’s important to note about the yuck factor, says Alvin Roth, a professor of economics and business administration at Harvard University, is that it’s often culturally based. So, while some concepts or objects are universally repugnant (feces, for instance, which researchers studying disgust call a “core disgust elicitor”), others are repugnant in some cultures but not in others. In the Summer 2007 *Journal of Economic Perspectives*, Roth wrote that California restaurants were banned by referendum from selling dog or horse meat because the majority of voters viewed their consumption by humans as repugnant. But throughout Africa and Asia, both these meats are as popular as hot dogs and hamburgers, and they routinely wind up on the dinner table.

Taking that analysis in a related direction, Dan Kahan, a professor at Yale Law School, argues that risk perceptions can vary at the individual level according to one’s views of how society should be structured. Kahan breaks society down into two personality types: individualists, who believe people should compete for resources and fend for themselves; and communitarians, who believe people should work together in a spirit of solidarity.

In that context, people of either group aim to protect their own social identity and interpret risks according to who communicates them, Kahan explains. Individualists tend to be risk-skeptical and unconvinced about threats raised by communitarians. Likewise, communitarians can be repelled by technologies imposed on the group by entities they deem untrustworthy.

When people are just beginning to learn about potentially controversial ideas, their reaction often depends on where their information comes from and how it is presented. For instance, an individualist might concede that anthropogenic climate change is a problem if the solution encourages more autonomy, deregulation, and business opportunity instead of just pollution controls mandated by authorities. Donald Braman, an associate professor at George Washington University Law School, adds that it’s possible to alter people’s reactions to a proposed threat merely by changing the source of the information about it. Thus, he says, a communitarian who was once repelled by the thought of drinking recycled wastewater might reverse that view if support for it comes from someone with shared cultural values.

## The Power of Words

Choices in language and terminology are pivotal in determining how new technologies are received, asserts Haddad. “Opponents to water reuse exploit the yuck factor by calling it ‘toilet-to-tap,’” he says. “That’s a direct appeal to defensive, visceral reactions; it creates discomfort in the community, and it puts proponents on their heels.”

Similarly, some focus groups have expressed distaste for the field of synthetic biology—which creates new life forms by assembling DNA structures in the laboratory—merely on the basis of what it’s called, according to Julia Moore, deputy director of the Project on Emerging Nanotechnologies at the Woodrow Wilson International Center for Scholars in Washington, DC. “In focus groups we found that upon hearing about synthetic biology [for the first time], people tended to form negative opinions about it,” she says. “It may be that in the last decade ‘synthetic’ has become a bad word, compared to ‘organic’ and ‘natural,’ which are good words. So, when you put ‘synthetic’ and ‘biology’ together, it raises concerns. That’s different from the term ‘nanotechnology,’ which sounds hip and trendy. ‘Synthetic biology’ immediately seems to conjure up threatening images, like cloning.”

For many people, especially those more educated, it is important to justify actions with good reasons, says Rozin. Thus, he says, people who strongly prefer “natural” foods claim the preference is based on greater safety, better taste, and more sustainable production. “However,” he adds, “our research shows that even if we can convince people that the commercial version of a food is as safe, good tasting, and friendly to the environment as the natural version, they still prefer the natural. In fact, for most people, ‘natural’ is inherently better. But to state that sounds arbitrary, so they invent reasonable explanations for their preference.”

Those trying to overcome the yuck factor with data have their work cut out for them. It’s easier to appeal to visceral sentiments with repugnant imagery, Haddad points out, than it is to appeal to cognitive perceptions with technical information. “Scientists have to communicate so that the public hears what they’re saying,” he says. “People just want to know: what does this mean, and how does it affect me?”

As for who delivers that message, Haddad and Rozin’s research shows the most trusted sources are government or university scientists who don’t have a stake in whether a technology gets adopted or not. The least trusted entities, according to Moore, are corporations. That dichotomy illustrates an important element in the yuck factor: When it comes to controversial technologies, skeptics care about economic winners and losers, particularly when the risk–benefit ratio seems vague and uncertain.

GM foods provide a ready example, says Fiona Fox, director of the London-based Science Media Centre, a nonprofit organization. The vast majority of genetic modification in food crops is meant to make the plants pest- and herbicide-resistant such that they require less capital input from industrial-scale farmers during production, says Fox. “British consumers saw economic benefits going to companies . . . but they didn’t see any real public benefits coming from GM food,” she explains. “They didn’t need more choice in the supermarket; in fact, they were already dazzled by food choices. So you had a situation in which uncertain benefits were compounded by the fear that comes from messing with nature. And opponents to GM food exploited that gut reaction by calling it ‘Frankenfood.’”

The net result, Fox says, is that most British supermarkets refuse to sell GM food. For a population such as the British, that’s not much of a problem, she asserts. But university scientists aiming to create drought-resistant GM crops for the developing world have also seen their funding dry up, she adds. “I don’t really care if the British say yes or no to GM food,” Fox says. “But I do care that during the public debate, gut reactions were exploited, and because of that consumers didn’t have the opportunity to decide according to evidence-based science.”

## Overcoming the Yuck Factor

The lesson to be learned from that experience, Moore says, is that a technology’s benefits should be stressed clearly from the outset. And the benefits most enticing to the public, she claims, are those that appeal to health improvements. “Those [technologies] designed to promote better-performing consumer products rate last,” she says.

Scientists have to communicate so that the public hears what they’re saying. People just want to know: what does this mean, and how does it affect me?

–Brent Haddad, University of California, Santa Cruz

But even health improvements may not be appealing enough to overcome the feelings of dread provoked by a technology. Synthetic biology, for instance, could offer new tools for studying the molecular basis of disease, among other beneficial applications, but scientists have also used it to create pathogens including the Spanish influenza virus, raising the specter of new ways to create biological weapons. Likewise, among its other benefits, nanotechnology could supply new and superior drug delivery systems, but at the expense of health and ecological risks that have yet to be acceptably characterized, according to Moore.

A long-standing question in the field of risk perception is the degree to which gut reactions against unsettling technologies have intrinsic value as warning signs. Leon Kass, a professor at the University of Chicago who chaired President George W. Bush’s Council on Bioethics from 2002 to 2005, and who led that administration’s efforts to ban embryonic stem cell research on moral grounds, wrote famously in a June 1997 essay published in *The New Republic* that “. . . in crucial cases, repugnance is the emotional expression of deep wisdom, beyond reason’s power to fully articulate it.” (Haddad agrees there’s wisdom in repugnance—pointing out that over millions of years, humans have instinctually learned to avoid grossly polluted water.) Kass built on that concept in his essay to say instinctual repugnance might correctly lead us to reject technologies that defy the “central core of our humanity,” citing human reproductive cloning as the quintessential example. “Shallow are the souls that have forgotten how to shudder,” he wrote.

Caplan, on the other hand, argues that “at its best, repugnance is a trigger that should lead you to ask why you feel a certain way about something.” He adds, “It should never be regarded as an end point or the basis of an argument. To me, a sophisticated examination of intuition is much more impressive. People used to think the idea of women voting or black people drinking from a ‘whites-only’ fountain was repugnant.”

To those confronted with hard choices over new and emerging technologies that may have profound implications for environmental and public health, Kahan’s advice is to seek input not just from like-minded people who share similar views but also from trusted sources who share an opposing view. That bipartisan approach would serve society well as science becomes ever more transformative. Indeed, a growing convergence of nanotechnology, synthetic biology, and genetics has introduced increasingly powerful applications. But, driven by a relentless exploration of what’s possible in science, society also has to consider what’s useful and necessary. While gut reaction can serve as a helpful guide in this process, we would be wise to remember the words of philosopher George Santayana, who wrote, “Well-bred instinct meets reason halfway.”

## Figures and Tables

**Figure:**